# A feast for *Candida*: Metabolic plasticity confers an edge for virulence

**DOI:** 10.1371/journal.ppat.1006144

**Published:** 2017-02-09

**Authors:** Pedro Miramón, Michael C. Lorenz

**Affiliations:** Department of Microbiology and Molecular Genetics, The University of Texas Health Science Center, Houston, Texas, United States of America; McGill University, CANADA

## Introduction

*Candida albicans* is an opportunistic fungus that can cause both mucosal and invasive infections. As a commensal, *C*. *albicans* is well adapted to the rapidly changing environments within the host, where availability of many nutrients is limited. Any organism trying to colonize these environments must adapt to the nutrients that are available in order to thrive and also must cope with mechanisms of immune surveillance. This Pearl will discuss recent findings highlighting the remarkable ability of *C*. *albicans* to utilize a wide array of nutrients and how this ability has been channeled to promote both commensal and pathogenic interactions.

## What nutrients are available in the host?

**Fig 1:**
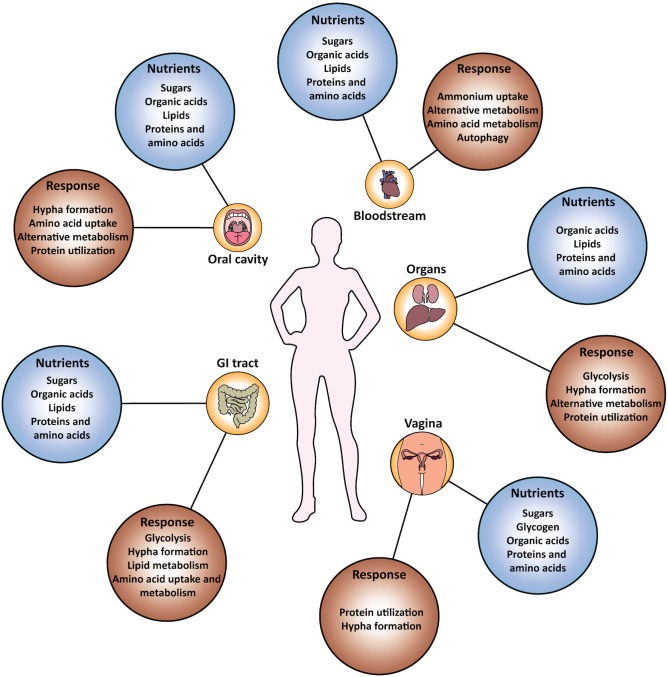
Available nutrients in infection-relevant niches and responses from *C*. *albicans*. The nutrients available in diverse niches are summarized. Note that the overall nutrient composition remains constant, but the response varies from one anatomical site to the other. “Alternative metabolism” refers to nutrients catabolized via pathways other than glycolysis, such as the glyoxylate cycle, fatty acid β-oxidation, and amino acid degradation. Key references: oral cavity [2,10], bloodstream [11–13,47], organs [15,19], gastrointestinal (GI) tract [4,48], vagina [1,49].

Nutrients available in the host come both directly from the diet and after processing by the combined metabolism of the host and microbiota (Fig 1). Simple sugars from the diet are rapidly absorbed in the small intestine and are available in the blood at low concentrations (0.1%–0.2%). Bacteria in the colon contribute to the digestion of complex carbohydrates and produce a variety of metabolites such as organic and short chain fatty acids (lactate, acetate, butyrate, propionate); lactate is particularly abundant. Salivary and vaginal fluids are also nutritionally complex and are rich in protein and protein byproducts (peptides and amino acids) [1,2]; the highest protein concentrations are found in the blood. Other carbon sources found in the host include the abundant amino sugar *N*-Acetyl-glucosamine (GlcNAc), a common constituent of glycosylated proteins from the host and a major component of bacterial cell wall peptidoglycan. Lipids and phospholipids found ubiquitously in cell membranes also represent a potentially accessible nutrient.

## How is *C*. *albicans* sensing the nutrients in the host?

**Fig 2:**
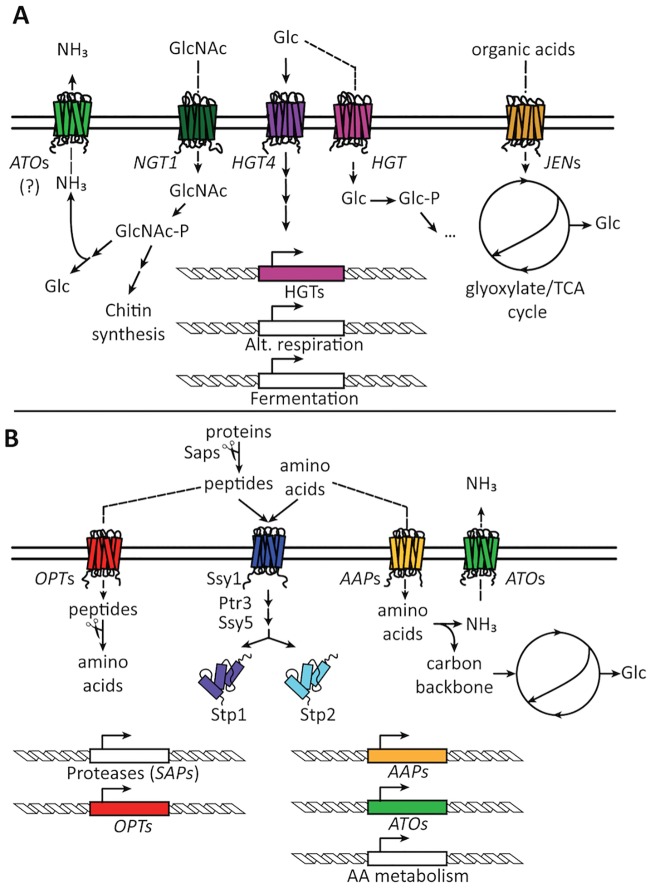
Sensing mechanism and transport systems for the utilization of different carbon sources. A) Sensing of monosaccharides via Hgt4 up-regulates the expression of sugar transporters and metabolic genes. Organic acid uptake is facilitated via Jen transporters. B) Sensing of peptides and amino acids via the Ssy1-Ptr3-Ssy5 (SPS) complex up-regulates the expression of amino acid permeases (AAPs) and oligopeptide transporters (OPTs) as well as secreted proteases and amino acid catabolic genes. Resulting ammonia from amino acid catabolism is extruded via ammonia transporters (ATOs).

Glucose remains the preferred carbon source (Fig 2A), and *C*. *albicans* detects this sugar via three sensing pathways that act in concert to activate glucose transport and metabolism: the sugar receptor–repressor pathway, the glucose repression pathway, and the adenylate cyclase pathway (reviewed in ref. [3]). Glucose metabolism via glycolysis and fermentation seems to be crucial during *C*. *albicans* gastrointestinal (GI) colonization, since genes controlled by the glycolysis transcriptional regulators Gal4 and Tye7 are highly expressed in the cecum of mice. Interestingly, whereas Tye7 is required for GI colonization, this transcription factor (TF) is dispensable for systemic infection [4]. In contrast, glycolytic enzymes are critical for systemic pathogenesis [5]. This suggests there are additional regulators yet to be discovered that control central metabolism in some host niches.

Amino acids are a preferred nitrogen source in most fungi, but only a few species have been reported to effectively utilize them as a sole source of carbon, with *Candida* species as a key exception [6,7]. Extracellular amino acids are sensed via the SPS system, a three-protein complex consisting of the permease-like sensor Ssy1, the relay protein Ptr3, and the endoprotease Ssy5. The SPS system proteolytically activates two transcription factors: Stp1, which controls the expression of extracellular proteases and peptide transporters, and Stp2, which controls the expression of amino acid permeases and catabolic enzymes [8,9]. The combined activity of the Stp proteins increases the supply, import, and catabolism of amino acids (Fig 2B).

These sensing mechanisms are highly interconnected and share common elements [3]. It is not unreasonable to think that these circuits have evolved in this pathogen to act in an orchestrated way in order to have a coordinated response depending on the nutrients that are available. How *C*. *albicans* senses other nutrients (e.g., lipids, phospholipids) is not well understood.

## Metabolic flexibility as a strategy for virulence

Evidence suggests that *C*. *albicans* utilizes multiple carbon sources in the host (Fig 2). During infection of organs and tissues, a signature shift in gene expression is seen towards alternative carbon utilization through the up-regulation of pathways, such as gluconeogenesis, glyoxylate cycle, and β-oxidation of fatty acids (Fig 1). This response has been observed in several infection models, such as reconstituted human oral epithelium (RHE) [10], murine macrophages [11] and human neutrophils [12,13].

*C*. *albicans* makes use of both sugar and nonsugar carbon sources during infection, and impairment of individual pathways reduces virulence and colonization [14]. Indeed, in contrast to *Saccharomyces cerevisiae*, data suggests that *C*. *albicans* will utilize diverse carbon sources simultaneously. Single-cell reporter strains provide evidence for active switching between glycolysis and gluconeogenesis at a transcriptional level [15]. While the addition of glucose triggers the rapid ubiquitin-dependent degradation of alternative assimilation pathways in *S*. *cerevisiae*, these same enzymes, including Icl1 (glyoxylate cycle) and Pck1 (gluconeogenesis), have specifically lost ubiquitination sites in *C*. *albicans* such that metabolism of lactate, notably, continues for extended times after glucose readdition [16,17]. The consequences of ubiquitination rewiring in these key pathways has an enormous impact on the ability of this fungus to colonize the GI of mice, to resist phagocyte attack, and in general to cause infection [18].

A second characteristic response is the induction of amino acid permeases, oligopeptide transporters, and amino acid catabolic genes, which can be seen during oral candidiasis, in cells phagocytosed by macrophages or neutrophils, and in tissue [10–12,19]. Moreover, amino acid auxotrophs in several species retain full virulence [20–22], reinforcing the idea that amino acids are readily available in the host. Vacuolar proteases are also induced, suggesting that *C*. *albicans* is catabolizing proteins acquired through endocytosis [11,12]. Mutants defective in autophagy are not more susceptible to macrophage killing than the wild type counterpart [23], which suggests that *C*. *albicans* finds a source of host protein to use.

*C*. *albicans* has an expanded family of ten secreted aspartic proteinases that mediate the utilization of host proteins and are involved in tissue invasion and pathogenicity [24,25]. Similarly, it encodes an expanded family of eight oligopeptide transporters [26] as well as many amino acid permeases [8]. This indicates that *C*. *albicans* has a very robust system for the utilization and uptake of host proteins.

In addition to these nonfermentable carbon sources, *C*. *albicans* can assimilate sugars that are not commonly utilized by other fungi, such as N-Acetyl-glucosamine (Fig 2A). The GlcNAc transporter and metabolic genes are induced during macrophage phagocytosis [11,27], and mutants unable to utilize GlcNAc are defective in both virulence and commensal persistence [28,29].

Lactate is abundant in many host niches, and *C*. *albicans* uses this nutrient as a signal to promote profound changes in its cell wall architecture that impact the resistance of this pathogen to multiple stresses [30]. Macrophage phagocytosis induces the expression of lactate transporters and metabolic enzymes [11,31], which suggests that this organic acid may be available inside the phagocyte. The carboxylic acid transporters Jen1 and Jen2 [32,33] are both induced upon phagocytosis, though they do not appear to be required for systemic virulence [33].

*C*. *albicans* secretes a wide array of lipases and phospholipases [34] that may have overlapping roles in nutrient acquisition and host damage. Secreted lipases have been associated with virulence in *C*. *parapsilosis* [35] as have phospholipases in *C*. *albicans* [36]. Fatty acid catabolism via the β–oxidation pathway feeds into central carbon pathways and is also required for full virulence [37].

## Quirks of metabolism that contribute to virulence

Nutrient metabolism directly affects host–pathogen interactions. As mentioned above, the presence of lactate influences resistance to osmotic and cell wall stress [30]. These cells are also less visible to the innate immune system, shifting cytokine production away from IL-17 (a key proinflammatory cytokine) and toward the anti-inflammatory cytokine IL-10. Additionally, lactate-grown cells are less efficiently phagocytosed by macrophages and more capable at escaping when they are engulfed [38].

Incorporation of amino acids into carbon metabolism starts with their deamination; the released amino group is extruded as ammonia [39,40], a strongly basic compound (Fig 2B). Amino acid catabolism appears particularly important in macrophages, in which *C*. *albicans* interferes with the normal acidification of the phagosome, inhibits the maturation of this organelle, and autoinduces hyphal morphogenesis, which contributes to the escape from (and death of) the macrophage by both physical disruption and induction of pyroptosis [39,41–43]. *C*. *albicans* also alters extracellular pH during growth on GlcNAc, which potently induces the switch to the hyphal form. Unlike with amino acids, catabolism of GlcNAc is not required for the induction of morphogenesis [44]. Thus, it has a signaling role in addition to being a nutrient, similar to that proposed for lactate. In each scenario, *C*. *albicans* is using nutritional signals to trigger adaptations to the host.

## Summary

*C*. *albicans* has not only an extraordinary flexibility to utilize a variety of nutrients present in the host but also the ability to manipulate the microniches in order to cause disease and avoid the immune system. We are only beginning to unravel the strategies that this yeast uses to modulate its environment, but it is clear that the presence of several carbon sources contributes to fitness both as nutrients and as signals to affect extracellular pH, stress resistance, and cell wall structure. For instance, our group has just described how *C*. *albicans* is able to robustly modulate the environmental pH in response to carboxylic acids [45], a phenomenon that contributes to resistance to phagocytic cells. Growth on one of these carboxylic acids, lactate, results in profound changes to the *C*. *albicans* cell wall by masking β-glucans, an effective immune evasion tactic [46]. Utilization of GlcNAc, a common sugar in host environments, modulates extracellular pH and induces hyphal growth [28,29]. Thus, there are many examples of how metabolism of specific nutrients directly impacts pathogenic behavior. Further understanding of these mechanisms will shed light on the strategies that underlie the commensal and pathogenic fitness of *C*. *albicans*.
